# Resilience of warm-season (C_4_) perennial grasses under challenging environmental and management conditions

**DOI:** 10.1093/af/vfad038

**Published:** 2023-10-13

**Authors:** Joao M B Vendramini, Maria L Silveira, Phillipe Moriel

**Affiliations:** Range Cattle Research and Education Center, University of Florida, Range Cattle Research and Education Center, Ona, FL 33865, USA; Range Cattle Research and Education Center, University of Florida, Range Cattle Research and Education Center, Ona, FL 33865, USA; Range Cattle Research and Education Center, University of Florida, Range Cattle Research and Education Center, Ona, FL 33865, USA

**Keywords:** climate change, forages, grazing, livestock, warm-season grasses

**Implications**

- Warm-season (C_4_) perennial grasses are the main source of nutrients for livestock in tropical and subtropical regions with warmer temperatures. Climate change and increasing global temperatures may favor the expansion of areas with C_4_ grasses, previously occupied by C_3_ plants.
- Although C_4_ grasses are known for their resilience under stressful defoliation management, differences in anatomical and morphological characteristics can affect the resilience of these species to management practices. In addition, N is usually the most limiting nutrient for C_4_ grasses, while growing and the combi nation of N supply along with defoliation intensity and frequency may dictate the expansion and perennation of different C_4_ grass species in newly populated areas.
- In addition to greater forage production, C_4_ grasses have potential to increase CO_2_ mitigation due to the efficient photosynthetic pathway. A greater proportion of the biomass accumulation in C_4_ grasses occurs below ground, increasing C concentration in the soil and potential C sequestration.
- In summary, the expansion of C_4_ grasses in the world due to climate change has potential to increase forage production for livestock and mitigate greenhouse gas emissions in agriculture systems.

## Distribution and Physiological Mechanisms of C_4_ grasses

**Figure 1.**
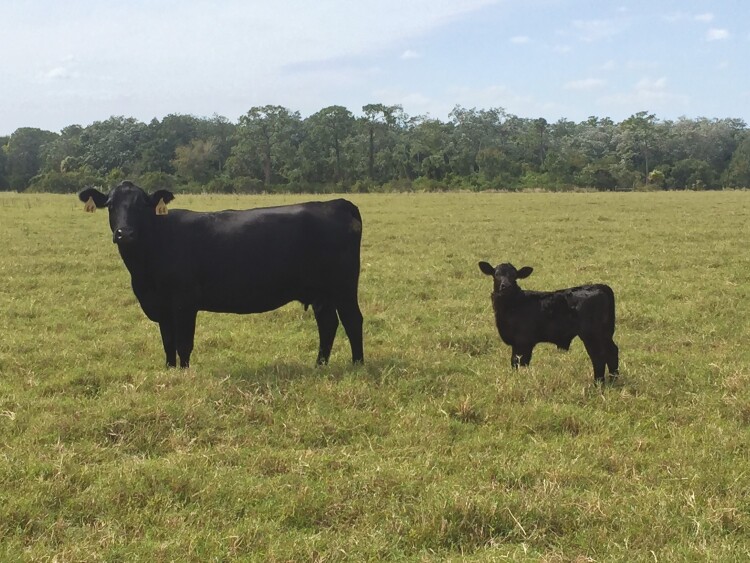
Cow-calf pair grazing ‘Mislevy’ bermudagrass [*Cynodon dactylon* (L.) Pers.] in Florida.

Warm-season (C_4_) perennial grasses are widely spread and dominant in tropical and subtropical regions, between 30^o^N and 30^o^S latitude, and it is perceived that more than two-thirds of all grasses in these regions are C_4_. The most extensive natural C_4_ biomes are the various savannas of the tropics, subtropics, and warm temperate areas that range in C_4_ grass cover from 20% to nearly pure C_4_ grassland (Cole, 1986; Figure 1). However, C_4_ grasses are spread in temperate regions and can be important forage resources for livestock during the growing season in those locations (Moser et al. 2004). According to Sage et al. (1999), at warm-temperate latitudes, C_4_ species dominate grassland productivity, even though they may represent less than 60% of the grass flora of the region. It is estimated that C_4_-dominated grasslands support approximately 40% of the world’s ruminant animals (Moser et al. 2004) and it is an important source of nutrients and shelter to several wildlife species in Africa, South America, North America, and Oceania (Sanderson et al., 2004).

Temperature has been the most significant factor dictating the presence or persistence of C_4_ grasses in a specific location and growing season. Temperature is highly correlated with C_4_ abundance along both latitude and elevation gradients. According to Long (1983), C_4_ plants are not dominant where growing season temperatures are less than an average of 16 °C and minimum mid-summer temperature average less than 8 °C. Increasing atmospheric temperatures (global warming) may favor the dominance of C_4_ species in different ecosystems, therefore, management practices may have to be adjusted accordingly. Cerling et al. (1997) observed that stable carbon isotopic data collected over the past 20 years document a worldwide expansion of C_4_ grasslands through the displacement of C_3_ vegetation during the Late Miocene and Pliocene. Corroborating with those findings, Wang et al. (2015) observed that increasing temperatures caused a shift from C_3_ to C_4_ species in the desert steppe in China, as indicated by the decrease in C_3_/C_4_ ratio due to warming temperatures over a 6-yr period. The expansion of C_4_ grasses in these areas may be detrimental to biodiversity, but it may not be avoidable due to the decline of the native vegetation and aggressive propagation mechanisms of C_4_ grasses.

The C_4_ carbon fixation pathway, identified by Hatch and Slack (1966) is responsible for the adaptability of warm-season grasses in locations with high temperatures worldwide. The environmental regions with predominantly C_4_ grasses range from wetlands to deserts and some of the least productive to the most productive ecosystems on the earth (Wedin, 2004). It was later observed that C_4_ species had different decarboxylation systems and were divided into three main groups based on differences in the activities of C_4_ decarboxilation enzymes, named NAD-Malic (Hatch and Kagawa, 1974), NADP-Malic (Berry et al., 1970), and PEP carboxykinase (PCK, Edwards et al., 1971). The three groups all have similar leaf cell anatomy; however, there are some structural variations, which appear to correlate with biochemical differences and may potentially impact the regions of adaptability of the main groups.

Dominant tall C_4_ grasses from different species are adapted to different regions of the world, such as big bluestem (*Sorghastrum nutans* L.) in North America, kangaroograss (*Themeda australis* (R.Br.) Stapf.) in northern Australia, thatchingrass (*Hyparrhenia* spp.) in South Africa, and bluestem (*Andropogon* spp.) in Brazil, which tend to be malate forming (NADP-Malic) enzyme variants. These locations are characterized by relatively high humidity and these grasses are usually managed at low-input levels (Vogel et al., 1978; Wedin et al., 2004). Taub (2000) compared the C_4_ grass flora and climatic records for 32 sites in the USA and found that the proportion of the grass flora that uses the NADP-Malic enzyme variant of C_4_ photosynthesis greatly increases with increasing annual precipitation, while the proportion using the NAD-Malic enzyme variant and PCK decreases. The NAD-Malic and PCK usually have greater growth potential and nutrient requirement, while the NADP-Malic group has slower growth rates, with greater cell wall deposition and efficient N use. Taub (2000) observed that there was a strong correlation between the frequency of the different subfamilies with annual precipitation that was independent of the influence of the different C_4_ variants. It therefore appears that other, as yet unidentified, characteristics that differ among grass subfamilies may be responsible for their differences in distribution across natural precipitation gradients and the association of grass subfamilies with annual precipitation was even stronger than for the C_4_ decarboxylation variants.

Besides the importance of the climatic conditions to different groups, soil fertility, and management conditions may interfere with the adaptability of C_4_ grasses with different enzyme variants to a specific location. Plant N use efficiency (NUE) has been a variable of interest, primarily in extensive grazing systems with low N inputs. The NUE may be due to variations in the efficiency of the photosynthetic apparatus, such as enzyme kinetics.

In general, it is expected that NADP-Malic plants have low late season forage N concentration (<5.0 g/kg), while aspartate forming (NAD-Malic and PCK) plants usually have greater tissue N concentrations (10–20 g/kg) (Wedin, 2004). LeCain and Morgan (1998) found that leaf N concentration was higher in three NAD-Malic grass species than in three NADP-Malic grasses, implying that NAD-Malic species may compensate for a lower NUE with greater allocation of N to photosynthetic organs. Bowman (1991) compared four NAD-Malic and two NADP-Malic members of the grass genus *Panicum* and found that at high levels of N availability the NAD-Malic species had higher shoot N concentrations than the NADP-Malic species. The NADP-Malic species may have a greater photosynthesis rate than NAD-Malic species at a given leaf N concentration. However, this trial compared subtypes within the same species and there may be larger inter-species variations.

The NAD-Malic plants may have relatively greater N requirement than NADP-Malic, primarily due to greater enzymatic steps and amino acids (Kanai and Edwards, 1999). Superior NUE of NADP-Malic relative to NAD-Malic grasses is achieved with less leaf N, soluble protein, and Rubisco having a faster turnover (Ghannoum et al., 2005). Lambers and Poorter (1992) hypothesized that low potential growth rates are associated with species found in N-poor habitats.

Recently, there has been identified genes related to NUE in crops. Genes that code for nitrate and ammonium transporters that assimilate N from the soil and genes that synthesize N compounds such as glutamine synthetase, which produces the amino acid glutamine (used to transport N through the plant) are examples of genes that have been recently evaluated to increase NUE in crops (Hirel at al., 2007). The identification and expression of these genes may be of great interest to forage crops, which are commonly cultivated under a limited supply of N fertilization; however, these preliminary studies have been conducted only in major crops, such as maize, rice, and wheat.

## Nitrogen Management in C_4_ Grasslands

Native and planted grasslands are frequently found in soils with limited production capability, many times in highly weathered, acid, and less fertile soils. Soil microorganisms, pH, structure, texture, organic matter, water, and many other characteristics influence forage growth, however, soil fertility is one of the most influential characteristics in forage production. The combination of low soil nutrient levels, efficient nutrient extraction capability, and high total yield potential create conditions for significant yield responses when grasslands are fertilized (Matthews et al., 2004).

Nitrogen is the most limiting plant nutrient for growth of grasses and yield and increasing crude protein (CP) responses have been observed with different species (Wilkinson and Langdale, 1974). Nitrogen has the greatest influence on forage yield and accordingly influences the amount of other nutrients required to sustain production at specific N levels (Taliaferro et al., 2004). Nitrogen is essential for amino acids and protein synthesis and formation of nucleic acids, which increase photosynthesis and forage production. The increase in DM production is frequently justified by the increase in tiller numbers, tiller weight, number of leaves per tiller, and leaf appearance rate in warm-season grasses (Moreira et al. 2009; Premazzi et al., 2003; Colozza et al., 2000). Premazzi et al. (2003) observed an increase in tiller number and weight on ‘Tifton 85’ bermudagrass (*Cynodon* spp.) fertilized from 0 to 160 mg N/kg and there was a significant correlation between tiller number and DM accumulation. Moreover, N fertilization also increases the number of leaves per tiller and leaf appearance rate. Garcez Neto et al. (2002) observed a quadratic increase in tiller number, leaf appearance, and leaf elongation rate of ‘Aruana’ guineagrass (*Panicum maximum* Jacq.). Leaf appearance rate ranged from 0 to 0.14 leaves/d and leaf elongation from 25 to 60 mm/d with N fertilization levels from 0 to 200 mg/dm^3^. Santos et al. (2009) observed that leaf elongation rate increased from 15.4 to 46.8 mm/d in ‘Marandu’ palisadegrass [*Brachiaria brizantha* (Hochst.) ex A. Rich] with N fertilization levels of 0 and 100 kg/ha, respectively.

It has been observed that N fertilization may not increase root-rhizome mass in warm-season forages (Alderman et al., 2011); however, it may affect carbohydrate reserve utilization. Nitrogen fertilization has decreased root-rhizome carbohydrate concentration of bermudagrass (Schmidt and Blaser, 1969). Root-rhizome mass and carbohydrate concentration are important factors involved in C_4_ grass regrowth and persistence; therefore, a combination of fertilization and adequate levels of defoliation frequency and intensity should be imposed to promote persistence of C_4_ grasslands. Liu et al (2011) observed that taller stubble heights promoted greater total nonstructural carbohydrate mass in Tifton 85 bermudagrass pastures grazed from 8 to 24 cm stubble height.

It is perceived that N fertilization may be a source of nutrient contaminant to waterways due to leaching and off-site movement; however, most C_4_ grasslands are managed extensively without or limited amounts of N fertilization, primarily due to economic constraints (Vendramini et al., 2014). In addition, C_4_ grasses efficiently uptake N and are used for phytoremediation of soils with increased nutrient concentration (Newman et al., 2009) and soil cover to decrease erosion and run-off (Sanderson et al., 2004).

## Defoliation Effects on C_4_ Grasses

Defoliation is a major determinant in C_4_ grassland productivity and persistence and it can be caused by fire, pests, mechanical harvest, or grazing. The resilience of the C_4_ grasses to defoliation is attributed to a combination of morphological and physiological characteristics.

Newly expanded grass leaves in the upper canopy are the primary site of photosynthesis, and as grass leaves age, they senesce and become less efficient in the use of solar energy for plant growth (Pitman, 1994). The upper leaves are usually the first tissues removed when the grassland is subjected to grazing, therefore decreasing primary production. Severe defoliation may remove photosynthetically active leaves, partially or completely, and carbohydrates stored in the reserve organs become the main substrate for energy for regrowth. It is observed that the most commonly used C_4_ grasses in subtropical areas have the ability to allocate photosynthate between reserve structures (rhizomes, stolons, and roots), and store C and N that can be remobilized after grazing, which are crucial for rapid regrowth after severe defoliation (Moore et al. 2004). If the defoliation is lenient, the plant will restore leaf area and photosynthesis will become the main supplier of energy. It is also noted that the most resilient grasses under grazing have meristematic zones positioned at, or below the soil surface, and they are therefore inaccessible to grazing animals. Additionally, the meristematic region is located at the base of the leaf, thus new leaf material can continue to grow even if older parts of the leaf are removed by grazing (Chapman and Lemaire, 1993). In addition to the robust reserve structures and protected meristematic points, some C_4_ grasses have avoidance mechanisms, which reduce the probability of defoliation of the plant by grazing animals. Those mechanisms can be morphological, such as stem elongation, thorns, etc. or antiquality chemical components.

According to Richard (1993), the degree of stress imposed depends on defoliation depends on frequency and intensity of defoliation, physiological age and type of removed tissue, and the occurrence of stress or competition before, during, or after the defoliation. There has been an extensive investigation on management practices to quantify optimum dynamics of leaf area expansion, light capture, and sward structural and compositional changes in C_4_ grasses (da Silva et al. 2015); however, research demonstrating persistence and productivity of C_4_ grasses under a stressful defoliation regime is scarce. This information is valuable because a great proportion of C_4_ grasses are cultivated under marginal management practices.

In general, new cultivars were selected and released over the last 50 years with the main objective to increase herbage accumulation and nutritive value, and this selection may have altered plant morphology, physiology, and preference by animals, which may negatively impact persistence. Vendramini et al. (2010) conducted a grazing trial to test the effects of different grazing frequencies on newly released cultivars of bahiagrass (“Tifton 9” and UF Riata) under a limited N fertilization regime. It was observed that Tifton 9 and UF Riata bahiagrass cultivars decreased root-rhizome mass and ground cover when grazed at 2 wk when compared to a 4 wk regrowth interval; however, Tifton 9 had greater root-rhizome mass than Argentine, Pensacola, and UF Riata when grazed at a 4 wk regrowth interval. It is recommended that new cultivars should be tested under a range of management practices, thus clear management guidelines are defined upon the release of the cultivar.

Stocking rate is the most common animal-based measure of grazing intensity and it is suggested that it is more important than any other single grazing management decision (Jones and Jones, 1997; Sollenberger et al. 2012). The main objective of numerous grazing intensity studies during the past five decades was to describe the form of the performance per animal curve as a function of stocking rate or grazing pressure (Sollenberger and Newman, 2007). Burns et al. (1989) compiled a series of grazing studies with fixed stocking rates and observed that average daily gain (ADG) is predominantly greater at low stocking rates. In low stocking rates, the animals have the option to ad libitum consumption of selected leaf components, which maximizes ADG. Increasing stocking rates may increase gain per hectare up to a limit where additional increase would result in limiting herbage mass and consequently reduction in ADG and gain per hectare (Mott and Lucas, 1952).

Although C_4_ grasses are known for their resilience under stressful defoliation management, few studies in the literature have reported the deleterious effects of grazing intensity and stocking rates on C_4_ grasses’ persistence. Rouquette et al. (2011) reported the effects of stocking rate on persistence of “Coastal” or common bermudagrass pastures with different fertilization and cool-season annual forage management and concluded that stands of Coastal and common bermudagrass were negatively affected by greater stocking rates and lack of N fertilization. Bahiagrass was the primary nonbermudagrass invasive species, while Coastal bermudagrass pastures grazed at greater stocking rates had a greater incidence of invasive bermudagrass ecotypes. Aguiar et al. (2014) observed that Jiggs bermudagrass pastures grazed at high stocking rates with average stubble height of 9 cm decreased Jiggs stands from 95% to 39% and increased invasive common bermudagrass from 4% to 36%.

Stocking method is a defined procedure or technique to manipulate animals in space and time to achieve a specific objective (Allen et al., 2011). Stocking methods are usually defined into two main categories: rotational or continuous stocking. The effects of stocking methods on resilience of C_4_ grasses have not been explored in research projects. Sollenberger et al. (2012) conducted a literature review and evaluated 27 publications that included rotational or continuous stocking treatments. Twenty-three publications (85%) showed an increase in forage quantity response for rotationally vs. continuously stocked pastures. According to Heitschmidt (1998), greater herbage accumulation occurs because the rotational stocking facilitates livestock distribution by increasing livestock density and reducing spatial variation in grazing pressure, which improves the harvest efficiency. It is hypothesized that rotational stocking allows better control of stubble height and pasture regrowth interval. As grazing intensity is a crucial component of long-term C_4_ grassland persistence, the choice of grazing method may not impact plant resilience, as long as the stubble height is maintained at the optimum levels for the specific C_4_ species. Sollenberger et al. (2012) concluded that “If conservation planning fails to identify, achieve, and maintain grazing intensity, then the choice of grazing method, season of grazing, and deferment, or any other grazing strategy will not be able to overcome this failure.” Forage and animal responses to stocking rates and methods are species and management specific and should be not extrapolated among different production systems, therefore, a long-term integrated approach to manage grassland should be identified for a particular grazing system.

## Greenhouse Gas Emissions Mitigation

Carbon stored in grazing lands represents 10% to 30% of world’s soil C stocks (Eswaran et al., 1993) and increase (or loss) of 1% of SOC sequestered in the top 10 cm of grazing land soils is equivalent to the total US agriculture emissions (Follett, 2001). The C_4_ grasses have potential to increase CO_2_ mitigation due to the efficient photosynthetic pathway and superior biomass accumulation. In addition, a greater proportion of the biomass accumulation in C_4_ grasses occurs below ground, increasing C concentration in the soil (Liu et al., 2011). The C_4_ grasslands produce 6% of the terrestrial global but have 15% of the global soil organic C (Jobbagy and Jackson, 2000). It has been observed that common management practices used to increase forage accumulation, nutritive value, and persistence can indirectly increase soil C and ecosystem services in grasslands (Silveira et al. 2013).

Adewopo et al. (2014) found that bahiagrass pastures fertilized with N had greater soil C content than bahiagrass with no N fertilization and rangelands in Florida. Despite the increase in C accumulation, it is perceived that N fertilization may increase N_2_O–N emissions, which would offset the benefits of greater soil C accumulation. However, many C_4_ grass grazing lands have extensive grazing systems with little or no fertilization. Garzon et al. (2022) observed that there was no difference in N_2_O–N emissions in bahiagrass plots fertilized with limited N fertilization levels (50 kg N/ha) and control (plots with no N fertilization). Approximately 7% of the N_2_O-N emissions in grasslands are from N fertilizer sources, while 54% are from animal excreta (Dangal et al., 2019).

In spite of the potential greater C assimilation, C_4_ grasses have usually greater cell wall and lignin concentration than C_3_ plants (Coleman et al., 2004), which may increase methane production in the rumen (Minson, 1990). However, several management practices can be used to decrease methane emissions by ruminants receiving C_4_ grasses, such as concentrate supplementation, inclusion of legumes in the pasture, and use of additives, such as tannins and ionophores, among others (Beck et al., 2018; Thompson and Rowntree, 2019; van Cleef et al., 2021).

**Figure 2.**
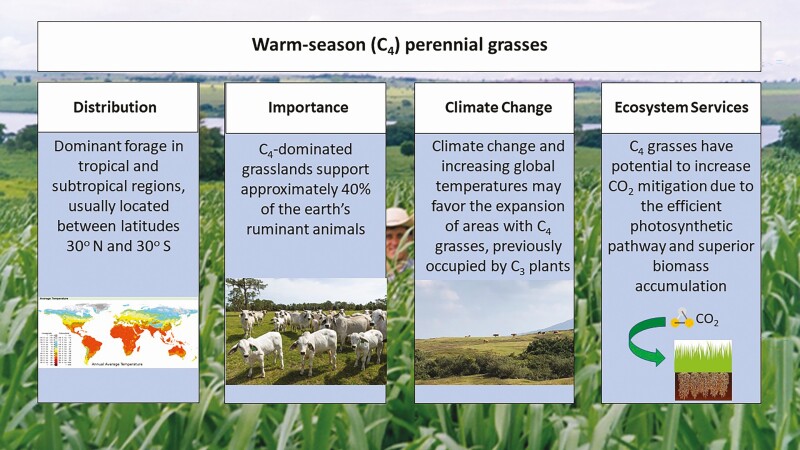
Warm-season (C4) perennial grasses distribution, importance, climate change, and ecosystem services considerations.

In summary, the rise in global temperatures favors the expansion of C_4_ grasses in regions previously occupied by C_3_ plants. This expansion is crucial to help to mitigate global warming due to C_4_ grasses’ efficient CO_2_ uptake and the ability to accumulate a greater proportion of the biomass below ground (Figure 2). Different photosynthetic pathways among C_4_ grasses may dictate the adaptability of the species to specific edaphic-climatic-management conditions. Proper soil nutrient and grazing management promote forage production, nutritive value, and persistence, in addition to enhancing C_4_ grasses’ greenhouse gas mitigation potential.

## About the Authors

**Philipe Moriel** obtained his master’s in animal science at the University of Wyoming in 2010 and Ph.D. in ruminant nutrition at the University of Florida in 2013. Currently, he is an Associate Professor at the Range Cattle Research & Education Center at the University of Florida and member of the Reproductive Task Force, and national and Southern sections of American Society of Animal Science. Philipe’s research and extension programs explore fetal and metabolic programming to develop nutritional strategies to optimize cow-calf production efficiency of *Bos indicus*-influenced cattle in subtropical/tropical environments.

**Joao Vendramini** received his BS degree in agronomy from the University of Sao Paulo, the MS degree in Animal Sciences from the same institution, and PhD in forage management at the University of Florida in 2005. He was Assistant Professor – Forage Specialist at Texas A&M University from August 2005 to August 2006 before taking his position at the UF/IFAS Range Cattle Research and Education Center, Ona, FL, where he is currently a Professor – Forage Specialist. His program is dedicated to forage management with emphasis on sub-tropical production systems. The major area of interest is forage-livestock interface and the impact of forage management on forage and animal production. He is a fellow of the Crop Science Society of America and a member of the American Society of Animal Sciences, American Registry of Animal Science Professionals, American Society of Agronomy, Crop Science Society of America, America Forage and Grassland Council, and Florida Cattlemen’s Association.

**Maria L. Silveira** is a professor of grassland biogeochemistry at the University of Florida (UF), Range Cattle Research and Education Center in Ona, FL. She earned her PhD degree in Soil Science from University of Sao Paulo, Brazil. Dr. Silveira joined UF in 2006 and her research is focused on nutrient management, soil and water conservation, and ecosystems services associated with cultivated and native grasslands. Through educational and outreach programs, she delivers science-based information to producers, extension faculty, industry professionals, and state regulatory agencies on topics related to nutrient management and pasture fertilization, soil health, and the role of pasture on soil carbon sequestration and greenhouse gas mitigation. She is a member of the Soil Science Society of America, America Society of Agronomy, Crop Science Society of America, and currently serves as Associate Editor for Agronomy Journal. She is also the chair of the USDA, multistate project W4170 “Soil-Based Use of Residuals, Wastewater and Reclaimed Water” and the director of the UF/Archbold Long-Term Agroecosystem Research (LTAR) Network site. Maria has received numerous awards including UF Research Foundation Professor, UF High Impact Publication, and Florida Cattlemen’s Association Research of the Year Award.
